# Strategy of developing nucleic acid-based universal monkeypox vaccine candidates

**DOI:** 10.3389/fimmu.2022.1050309

**Published:** 2022-10-27

**Authors:** Dimitri Papukashvili, Nino Rcheulishvili, Cong Liu, Xingyun Wang, Yunjiao He, Peng George Wang

**Affiliations:** Department of Pharmacology, School of Medicine, Southern University of Science and Technology, Shenzhen, China

**Keywords:** monkeypox virus, MPXV, mRNA vaccine, DNA vaccine, universal vaccine, pandemic

## Abstract

Until May 2022, zoonotic infectious disease monkeypox (MPX) caused by the monkeypox virus (MPXV) was one of the forgotten viruses considered to be geographically limited in African countries even though few cases outside of Africa were identified. Central and West African countries are known to be endemic for MPXV. However, since the number of human MPX cases has rapidly increased outside of Africa the global interest in this virus has markedly grown. The majority of infected people with MPXV have never been vaccinated against smallpox virus. Noteworthily, the MPXV spreads fast in men who have sex with men (MSM). Preventive measures against MPXV are essential to be taken, indeed, vaccination is the key. Due to the antigenic similarities, the smallpox vaccine is efficient against MPXV. Nevertheless, there is no specific MPXV vaccine until now. Nucleic acid vaccines deserve special attention since the emergency approval of two messenger RNA (mRNA)-based coronavirus disease 2019 (COVID-19) vaccines in 2020. This milestone in vaccinology has opened a new platform for developing more mRNA- or DNA-based vaccines. Certainly, this type of vaccine has a number of advantages including time- and cost-effectiveness over conventional vaccines. The platform of nucleic acid-based vaccines gives humankind a huge opportunity. Ultimately, there is a strong need for developing a universal vaccine against MPXV. This review will shed the light on the strategies for developing nucleic acid vaccines against MPXV in a timely manner. Consequently, developing nucleic acid-based vaccines may alleviate the global threat against MPXV.

## Introduction

Widely neglected disease– monkeypox (MPX) deserves remarkable attention since it crossed African borders and cases have increased fast. MPX is a zoonotic infectious disease characterized by smallpox-like symptoms but with less severity and a lower fatality rate. Monkeypox virus (MPXV) belongs to the *Orthopoxvirus* genus in the family of *Poxviridae* and has two clades– West and Central African clades. Remarkably, the Central African clade has more severe outcomes and a higher mortality rate, up to 11% (1, 2). Furthermore, it is more severe in younger children where the mortality rate can reach up to 15% (1, 3). MPXV that is spread outside of Africa represents the West African clade which has milder symptoms and a significantly lower mortality rate, ~1-3.6% (2–4). Besides, the recent outbreak demonstrated that the virus causes more mortality in developing endemic countries compared with developed countries probably because of the less capable health system (2). MPXV was first identified in macaque monkeys in a Denmark research facility (5). Since then, this infectious disease is called “MPX”. However, the natural hosts of this virus are considered to be rodents (6).

**Figure 1:**
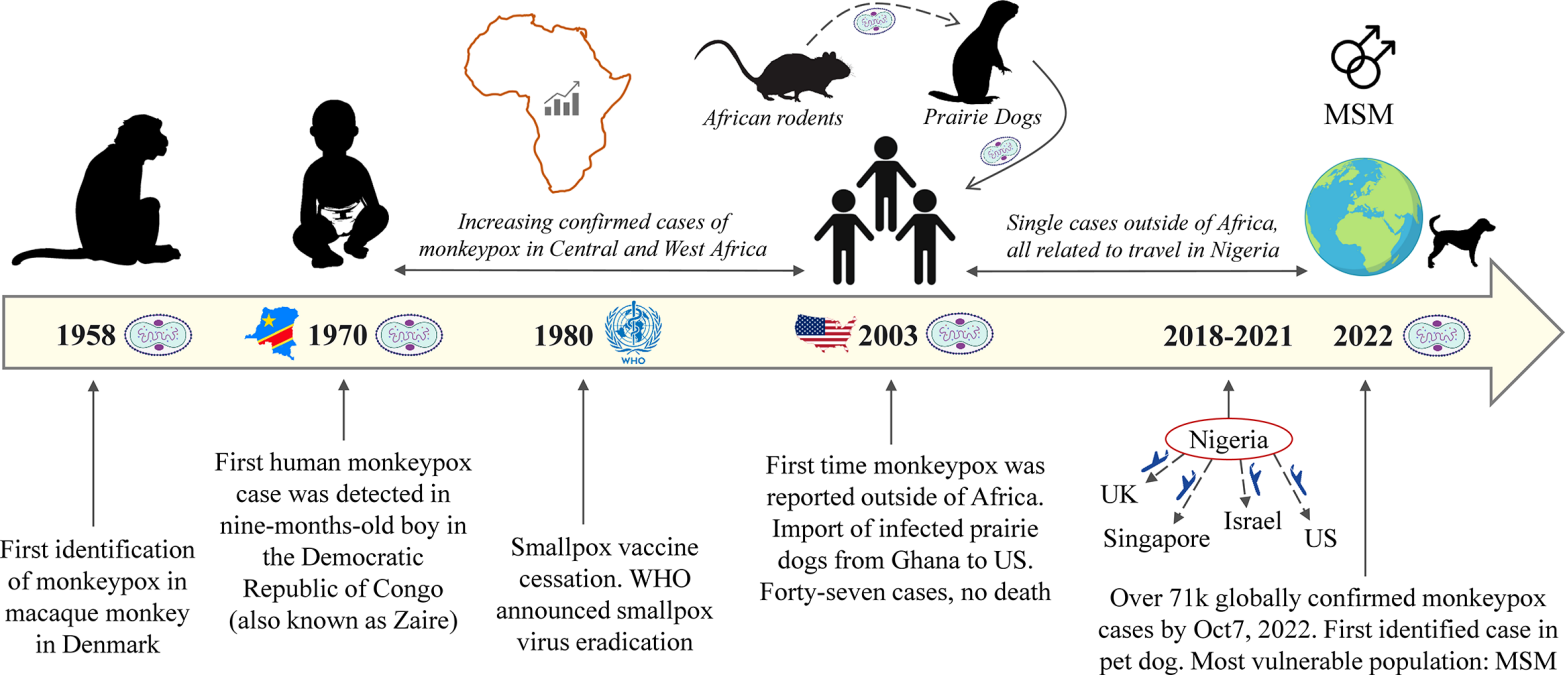
Timeline of MPXV from the first identification until the current outbreak.

The first human case was reported in 1970 during the active smallpox surveillance in Bokenda, a village in the Democratic Republic of Congo (DRC) (also called Zaire). The infected patient was a 9-month-old child whose sample was sent to the World Health Organization (WHO) Smallpox Reference Centre in Moscow and eventually revealed MPXV by isolation. Interestingly, the patient’s family admitted that monkeys were eaten occasionally as a delicacy but they were not able to recall if it happened during the last month or whether the child had any contact with the monkey in recent times (7). Importantly, the boy was not vaccinated against smallpox unlike the other members of the family (8). After that, MPX outbreaks mainly occurred in West and Central Africa until 2003, following the import of infected prairie dogs from Ghana to the United States (US). Remarkably, those dogs were housed or transported together with African rodents from Ghana. In total, 47 MPX cases were reported with no death (9, 10). In 2021, two cases of MPXV were reported in the US, both imported from Nigeria (11, 12). Apart from the US, few single MPX cases were reported in different countries such as the United Kingdom (UK), Israel, and Singapore (3, 13–16). All were related to travel to Africa, particularly Nigeria. However, since May 2022, the confirmed cases of MPXV dramatically increased outside of Africa, and currently, it is a global concern. WHO declared the MPX outbreak a global health emergency on July 23, 2022 (17). The timeline of MPXV spread since 1958 is given in **Figure 1**.

MPXV represents a linear double-stranded DNA (dsDNA) virus and it has two forms like other poxviruses– intracellular mature virus (IMV) and extracellular enveloped virus (EEV). MPXV has a large DNA genome (18) and is quite stable in the environment as DNA viruses mutate less frequently than RNA viruses (5, 19). Until 2022, the spread of the virus *via* secondary transmission outside Africa was limited (20). The current non-endemic MPXV outbreak is the first and largest outbreak to date that is different from the previous waves as an intensive human-to-human transmission takes place (3). The MPXV can be transmitted *via* exposure sources such as scratches and bites from an infected animal, saliva, respiratory excretions, contact with the lesion exudate, or even feces (8, 21). Except for the similarities to the symptoms of smallpox, unlike it, the clinical manifestation of MPX also includes lymphadenopathy. The rash appears 1 to 3 days after the onset of fever and lymph node enlargement. It can be distributed all over the body but mainly concentrated on the extremities (8), genitals, and anus (3, 22–25).

Despite the availability of a smallpox vaccine that has high efficiency to MPXV, there is no specific vaccine for MPXV. Hence, it is urgent to develop a strategy for designing a universal MPXV vaccine for further unexpected epidemics/pandemics preparedness. Nucleic acid vaccines, particularly mRNA vaccines have elicited high efficiency against ongoing coronavirus disease 2019 (COVID-19) pandemic (26–31). Moreover, DNA vaccines also show great results (32–36), e.g., India has approved a DNA vaccine against severe acute respiratory syndrome coronavirus 2 (SARS-CoV-2) (37). Nucleic acid vaccines have a number of advantages compared with traditional vaccines. Current review addresses the MPXV classification, structure, pathogenesis, and the strategy for designing nucleic acid-based universal vaccine candidates against MPXV.

## Monkeypox viruses– classification and structure

Along with MPXV, the genus *Orthopoxvirus* comprises three more human-pathogen species: variola virus (VARV)– the causative agent of smallpox, vaccinia virus (VACV), and cowpox. VARV and MPXV often cause life-threatening diseases, while VACV and cowpox are usually associated with local lesions. Out of two clades, the West African clade of MPXVs is characterized with less antigenic drift and virulence (38, 39). The Central African clade (clade 1) which is particularly endemic to the Congo Basin causes more severe symptoms of the disease as it is more virulent and transmissible (40, 41). The MPXVs isolated since 2017 are categorized as a clade 3 which along with clade 2 belongs to the West African clade (41, 42). MPXVs identified during the 2017/2019 outbreaks belong to lineages A.1, A.2, and A1.1, while MPXVs isolated during the current multi-country outbreak belong to lineage B.1 (41, 43). Importantly, clade 3 is characterized by the high number of mutations allowing increasing the adaptability to humans (42).

**Figure 2:**
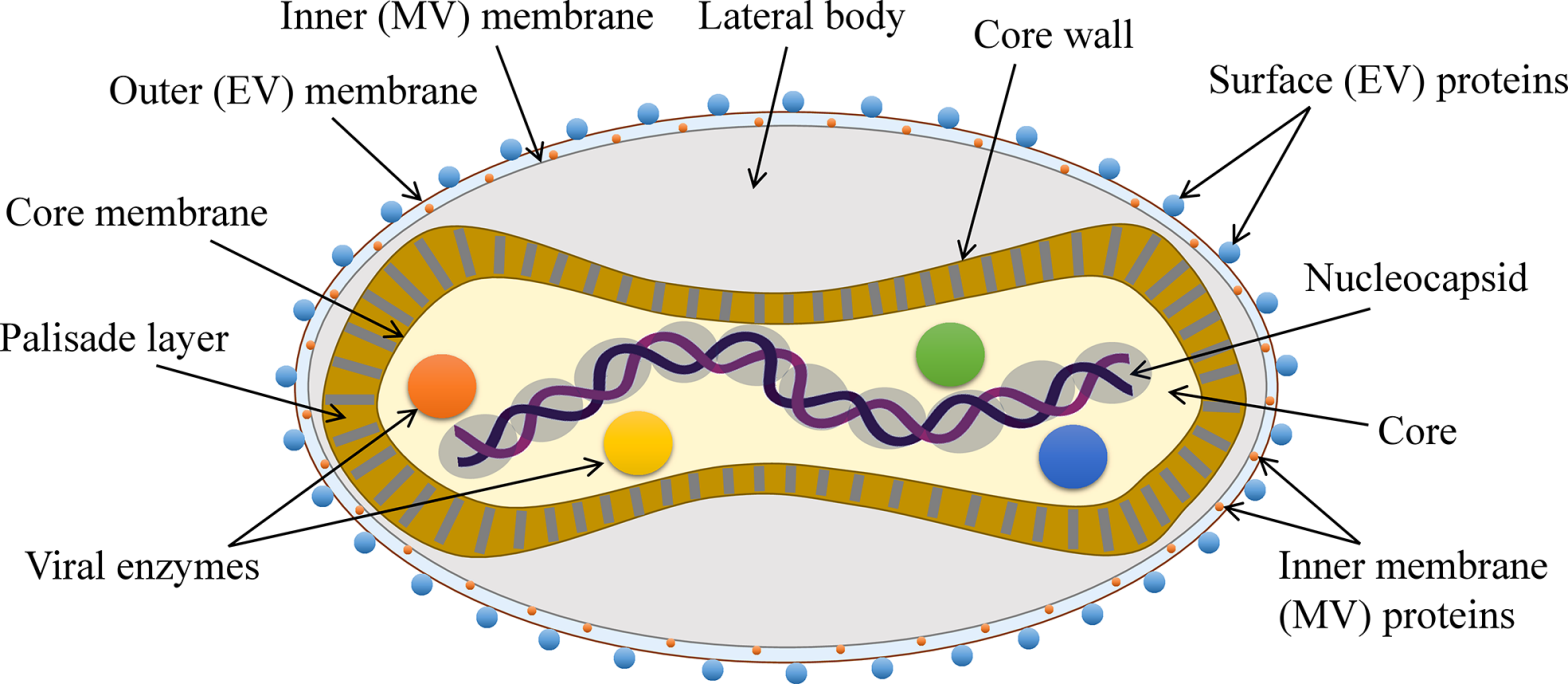
The general structure of MPXV.

MPXV like other poxviruses is a large (~280 nm X ~220 nm) (13), brick- or oval-shaped enveloped virus. The viral core is dumbbell-shaped and contains the enzymes necessary for uncoating and replication as well as the large ~197 kb long viral genome that is a linear dsDNA comprising over 190 open reading frames (ORFs) (3, 18). The MPXV has a complex structure and its genome is not fully characterized. Although there are at least 90 essential ORFs, most of the ORFs still need to be identified and studied (3, 44) (**Figure 2**). Like other poxviruses, MPXV also has two forms–EEV and IMV. EEV has an additional outer membrane and is considered to play a major role in early dissemination while IMV is released during the cell lysis. Both forms induce the infection (45, 46).

## Infection, pathogenesis, and clinical manifestation

After MPXV transmission through contact with an infected animal, human, or contaminated objects, the virus enters the body, disseminates systematically *via* monocytic cells, and can infect most mammalian cells (47). According to the clinical studies, lymphoid tissues in the neck and throat represent the primary replication areas for MPXV. After the primary lymphatic dissemination of the virus, liver and spleen are the major targets for the infection. The spread of the virus into small dermal blood vessels gives rise to the skin infection and lesions (48). The extracellular proteins of the poxviruses attach the glycans (laminin, heparin, and chondroitin sulfates) of host cells (49–51). H3L (heparan-binding surface IMV membrane protein), A29L (heparan-binding IMV surface membrane fusion protein), and E8L (chondroitin sulfate-binding IMV surface membrane adsorption protein) are among the proteins that are responsible for the attachment (49, 51). After the pH-dependent fusion and entry into the host cell, viral transcription takes place. Notably, transcription occurs *via* the viral DNA-dependent RNA polymerase. Hence, unlike other DNA viruses, MPXV does not need to be transported into the nucleus, instead, with its own machinery, the transcription takes place in the cytoplasm. Following transcription, translation occurs on the ribosomes of the host cell (52, 53). The majority of IMVs remain intracellularly and are released only upon the cell lysis while some of them become enveloped (intracellular enveloped virus (IEV)) by the additional outer membranes derived by the endoplasmic reticulum or Golgi apparatus. After the MPXV gains an additional membrane, it is either transported into the neighbor cell or outside the cell and becomes EEV (54). It is known that EEV infects the cells more efficiently compared with the IMV (55). Usually, the incubation period of MPXV lasts for approximately two weeks (56) and typically it is resolved within 2-4 weeks (22).

**Figure 3:**
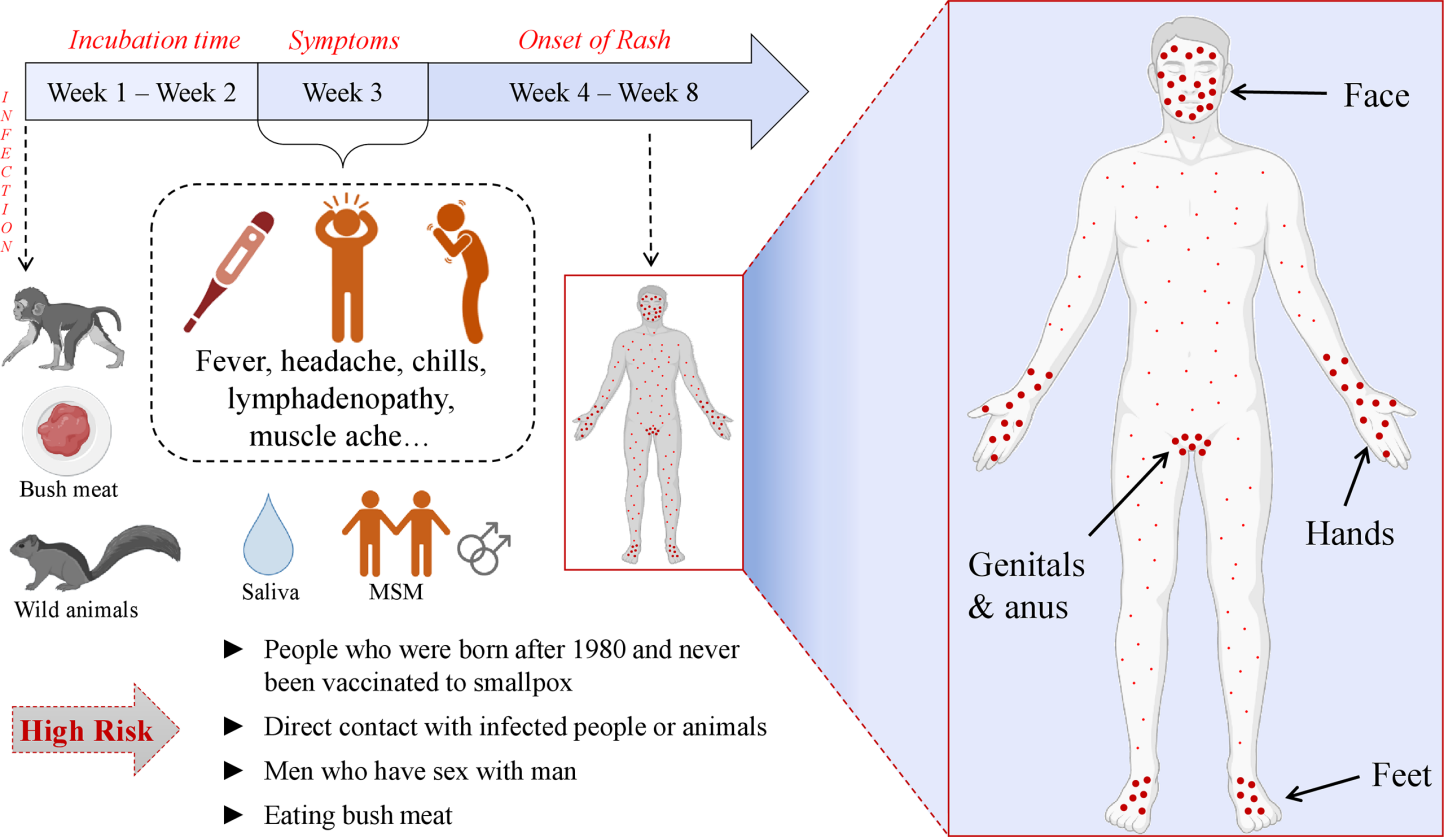
Pathogenesis and clinical manifestation of MPXV.

The MPX is characterized by similar symptoms as other poxviruses along with certain distinctive features. The common symptoms include fever, chills, body- and headaches, fatigue, sore throat, and rash that becomes papules and crust later while healing. Because of these similarities, MPX is often misdiagnosed with other poxviral diseases. The main difference between MPX and other poxvirus disease manifestations is that MPXV infection causes enlargement of lymph nodes before the development of rash (57–61). The rash is presented all over the body, usually concentrated on the face and extremities, however, the current multi-country outbreak of human MPX demonstrated the new tendency of atypical presentation. During the 2022 MPXV outbreak the lesions are usually localized in the genitals and/or anus of the infected patients (3, 22–25) and patients experience extreme rectal pain and penile oedema (62, 63). The complications of the MPX disease may be even life-threatening, e.g., encephalitis, sepsis, etc. (22). Besides, MPXV can be vertically transmitted making pregnant women and fetus vulnerable (48). Unfortunately, the lack of surveillance and health care in countries of Africa greatly contributes to the underdiagnoses of MPX and the spread of the virus, meaning that the numbers of daily cases have been more likely much higher than the officially recorded numbers (2). Notably, during the current outbreak, more MPX cases are detected in men who have sex with men (MSM) (62, 64–67). When the MPX cases were identified in Africa before spreading the virus outside the continent, it was notable that more confirmed cases were male. E.g., When 760 cases were detected in DRC during 2005-2007 through the surveillance program, the male patients (62.1%) predominated females (68). Martinez et al. have revealed that in Spain, a region of Madrid, by June 2022, 508 MPX cases were identified out of which 99% were men. 84.1% of the total number of cases had condomless sexual intercourse with multiple partners before the onset of the MPX symptoms. 93% of them were men who had sex with men. The distribution of the rash indicates that this type of close physical contact plays a major role in disease transmission (69). Remarkably, the tendency of smallpox vaccination among MSM is increasing (70). The illustration of MPX symptoms is given in **Figure 3**.

## Reasonings of 2022 MPX outbreak

On the one hand, the global concern has increased as MPXV crossed the African borders and cases have been increased fast, especially, since May 2022. However, it should not be considered an unexpected and unpredicted outbreak as there were many warning signs before MPX spread worldwide. Before the current outbreak took place, there was some favorable basis for MPXV to be disseminated worldwide. Noteworthily, a few months earlier before MPXV turned out into the center of global attention, Bunge et al. systematically reviewed human MPX epidemiology changes. They summarized that MPX cases were escalating, especially in DRC but it was also spreading to other countries, and besides, the median age was growing from young children to young adults (71).

**Figure 4:**
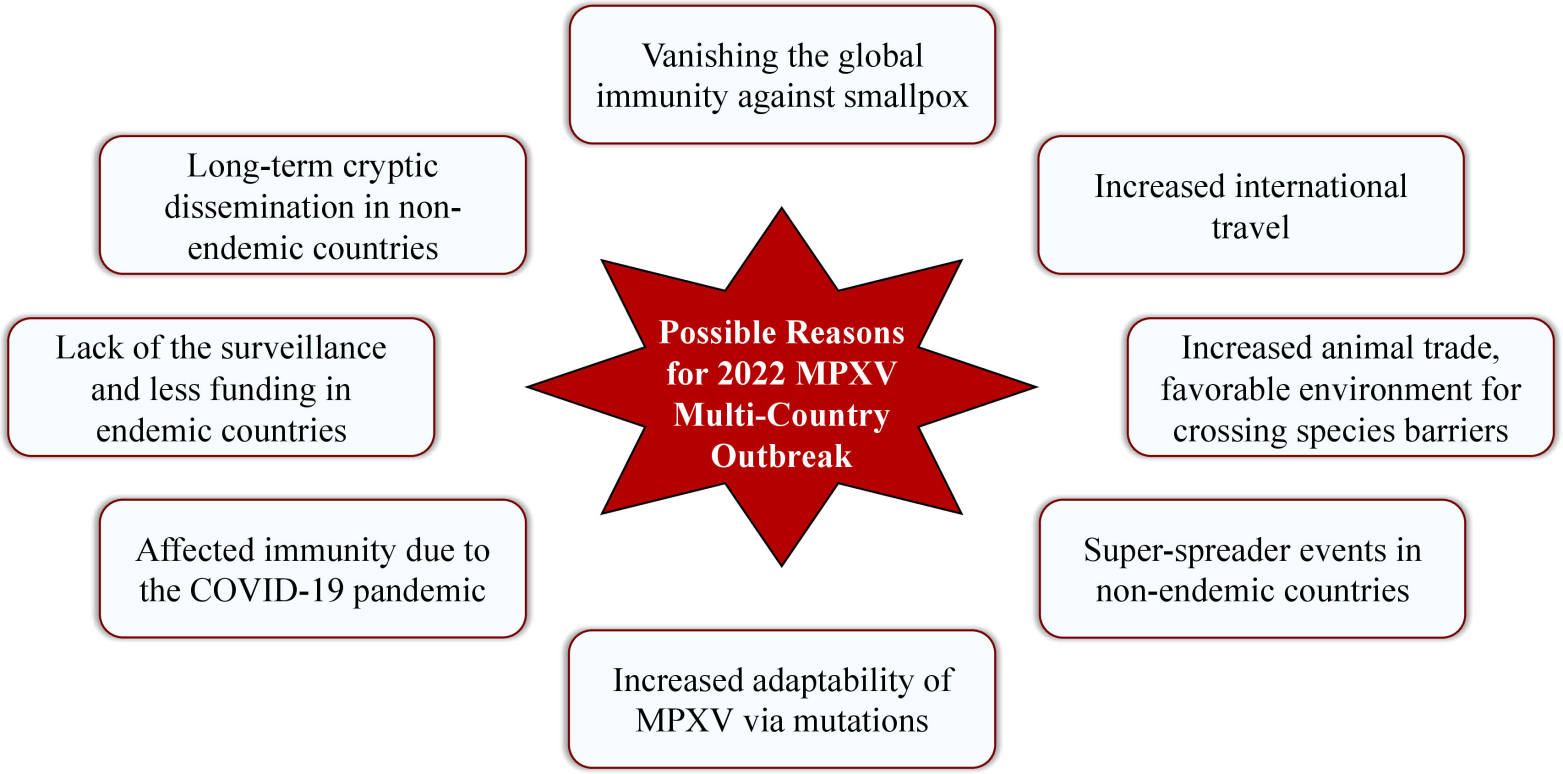
Possible reasons for 2022 MPXV multi-country outbreak.

There are several possible reasons that laid the groundwork for spreading MPXV worldwide in an unusual manner since May 2022. Some of the reasonable assumptions are: increased animal trade and making a favorable environment for crossing species barriers (8); increased international travel (3); long-term cryptic dissemination of MPXV in humans and animals in non-indigenous countries (42) along with the lack of the surveillance programs and less funding for health care in endemic countries (8); introduction of the virus in non-endemic countries by the super-spreader events (42); vanishing the global immunity against smallpox due to the eradication of smallpox infection and vaccination cessation since 1980 (5, 8, 72, 73) and acquiring the clinical relevance of MPXV (74); affected immunity due to the COVID-19 pandemic along with the increased adaptability of MPXV *via* increased mutation rate. The possible reasons for 2022 MPXV multi-country outbreak are illustrated in **Figure 4**.

As the smallpox vaccine is also effective (up to 85%) (74) in preventing MPXV and today approximately 70% of the world population is unvaccinated to smallpox and lacks vaccine-derived immunity (3), this reason seems to be one of the most rational for the current MPXV outbreak. Moreover, most of the MPX patients are under 50 and have never been vaccinated against smallpox. Interestingly, Adler et al. performed a retrospective observational study and found that there were 7 MPX patients registered in the UK from 2018 to 2021. Four of them acquired the disease outside, while 3 of them were in the UK. Out of these 3 patients, 2 developed the symptoms after arriving from Nigeria while the third patient was a healthcare worker who got MPX symptoms 18 days after contact with one of the other patients while taking care. More importantly, the third patient was vaccinated against smallpox (56). This also underlines the fidelity of long-term cryptic dissemination of the MPXV before the 2022 outbreak. All the abovementioned reasons indicate that the 2022 MPX outbreak was expected to occur.

## Prevention and treatment

The genome sequence of currently spread MPXV was reported on May 19, 2022, and was identified as a virus of the West African clade (67). Although this clade is characterized by less mortality and milder forms of the disease (75), it can still cause considerable morbidity and mortality. MPXV is still not a sufficiently studied virus and the treatment and prevention strategies are based on the methods used for the treatment of other Orthopoxviruses such as smallpox. E.g., Matias et al. have demonstrated that a pan-*Orthopoxvirus* inhibitor tecovirimat (TPOXX) which is approved by food and drug administration (FDA) for treatment of smallpox (76) could treat hospitalized MPX patients (77). Alternatively, there is another FDA-approved drug brincidofovir for the treatment of smallpox which is under investigation and has already shown efficacy against MPX in MPXV prairie dog models (78, 79). Additionally, cidofivir has demonstrated efficacy against Orthopoxviruses *in vitro* and *in vivo* studies (80–82). As the VAC immune globulin intravenous (VIGIV) is licensed by FDA for the treatment of complications caused by VAC vaccination, it is also considered one of the reasonable drug targets against MPXV for investigation (82). Currently available vaccines are JYNNEOS (VACV-based) and ACAM2000 (VACV-based) that are originally developed against the smallpox virus (83, 84).

## From variolation to nucleic acid vaccines

In 1774, the time when smallpox was spread, in the UK, farmer Benjamin Jesty was the first to immunize his family with material obtained from the cattle infected with cowpox (animal virus) to protect from smallpox (deadly human virus). As a result, all of them stayed healthy despite the numerous exposure to the smallpox virus (85). In 1796, key advancement in vaccinology took place. In the UK, Edward Jenner used a substance taken from the cowpox lesions of young dairymaid to inoculate an 8-year-old boy named James Phipps. Later, Edward Jenner inoculated the boy with the substance obtained from a fresh smallpox lesion and no disease was developed (85). As the word for a cow in Latin is “Vacca” and cowpox was used for the immunization against smallpox, Edward Jenner called this immunization procedure vaccination (85). He was the first person to confer scientific status on this new procedure and pave the way for the following scientific studies. This was the historic origin of immunization even though viruses as the causative agents of diseases were not yet identified (86). Smallpox which killed over 300 million people in the 20^th^ century was finally eradicated thanks to Edward Jenner’s enormous contribution (86). Interestingly, before this milestone, another turning point that had laid the groundwork for the development of immunization was variolation (inoculation)– an ancient Asian technique of infecting people with fluid from the smallpox pustules of a patient with a mild form of the disease. This strategy was also introduced in Europe, particularly, in the UK where Edward Jenner had experienced variolation himself when he was a little boy (87, 88). Several decades after the development of the first vaccine against smallpox (1798), in 1885, Louis Pasteur developed an inactivated virus vaccine against rabies (89). This is when the era of vaccinology and preventive medicine was launched. Anti-rabies vaccine continues saving millions of potential victims globally. Later in 1937, Max Theiler worked on the attenuation of the yellow fever virus and laid the groundwork for using live attenuated viruses for immunization. It was followed by the development of a series of live attenuated vaccines such as measles, rubella, varicella, etc. (86).

World scientists have kept advancing the prophylactic and therapeutic vaccinology all the time and the morbidity and mortality caused by various infectious diseases kept decreasing. Except for the physically or chemically inactivated and live attenuated vaccines, subunit vaccines comprising purified antigens, toxoid vaccines (inactivated bacterial toxins), as well as nucleic acid vaccines have been developed (90). Indeed, since the COVID-19 global pandemic has emerged, the new epoch of next-generation vaccines has begun. Evidently, the mRNA-based approach is advantageous owing to its high efficacy, safety, rapid development, low-cost, and cell-free manufacturing (26, 27, 91). In 1987 a key experiment was done by Robert Malone when he mixed mRNA with the synthetic cationic lipid incorporated into the liposome resulting in the successful transfection into the NIH 3T3 mouse cells and expression (92). Later in 1997 biochemist Katalin Kariko and immunologist Drew Weissman worked on the development of a human immunodeficiency virus (HIV) vaccine based on mRNA technology but as a result, a strong inflammatory response was observed in mice. Hence, they worked on the nucleoside modification and their approach was successful as mRNA was capable to escape innate immune response and increase the translation efficacy (93). Ultimately, the effective mRNA vaccines against COVID-19 that have emergency authorization– BNT162b2 (developed by BioNTech/Pfizer) and mRNA-1273 (developed by Moderna), contain the modified nucleobase N1-methylpseudouridines (m1Ψs) that modulates immune evasion, protein production, and effectiveness (94).

Along with the mRNA, DNA vaccines should not be forgotten as well. Both nucleic acid vaccines carry genetic information of the viral antigen into the host cells and allow them to produce the corresponding protein. This helps the body to develop immunological memory and to fight the real virus in a timely and effective manner in case of exposure (90, 95). mRNA needs to be delivered in the cytoplasm of the host cell to be translated into the target protein while DNA vaccine needs to be transported into the nucleus where it will be transcribed and after translocation of mRNA into the cytoplasm translation into the protein will take place (96). On the other hand, the time of DNA vaccine development from the design to commercialization is shorter (33). DNA vaccines, are considered to be safe (97), and compared to mRNA vaccines they elicit more stability at ambient temperatures (98). There are already approved DNA vaccines for veterinary use against highly pathogenic H5N1 influenza A virus in poultry (98), West Nile virus in horses, etc. (99). Same as in mRNA vaccines, the first human DNA vaccine was also approved for SARS-CoV-2 for emergency use in India (37). Additionally, there are a number of DNA (37, 100) and mRNA (101, 102) vaccines in clinical development.

## Need for vaccine development

For many years MPXV was considered to be geographically limited and it was not the center of foci for scientists. It recently re-emerged as the cases increased rapidly outside of Africa. This should be a wake-up call for other viruses as well, such as Crimean-Congo hemorrhagic fever (CCHF) virus, Zika, Ebola, etc. Fortunately, out of two major clades of MPXV, the West African clade is spread which is less severe compared with the Central African clade. Interestingly, the fatality rates for these two clades have a big difference as it is mentioned above (71). Nevertheless, we are not secure that the Central African clade of MPXV will never become an unpredictable deadly pandemic. It is also noteworthy that the MPX outbreak in Nigeria (West Africa) during 2017-2018 reported 122 confirmed cases with 7 deaths out of whom 4 were HIV-positive with poor disease control (103). Gay or bisexual men should be more careful and it is recommended for them to take active preventive measures such as vaccination. Even though it is reported that the smallpox vaccine provides protection against MPXV (104, 105), cases of MPX disease manifestation in smallpox-vaccinated patients are still observed (106). Moreover, rare but serious side effects such as myocarditis and pericarditis are still reported and certain groups of population are vulnerable to vaccination. Besides, the first and the second doses of JYNNEOS are administered 28 days apart. As it is unclear whether the first dose is enough for the sufficient efficacy, in case of post-exposure vaccination, 28 days is too late to protect from the MPX disease. Importantly, the risks of creating new virus strains *via* exchanging genetic information when infected with MPXV in vaccinated individuals is not understood. Moreover, the rate of recombination of the genes from live or attenuated poxvirus-based vaccines with MPXV is unknown (48). Hence, the specific, truly universal vaccine development for MPXV is essential. Additionally, the case of human-to-dog transmission of MPXV has already been detected which makes the eradication of the outbreak even more difficult (107). Moreover, if we observe the rationale of the tendency of infection outbreaks, we should appreciate the danger of other relatively forgotten viruses such as smallpox that has been already eradicated. If the MPXV emerged because of the wanned immunity against smallpox, smallpox itself with a mortality rate of 10-75% (108) might also re-emerge with a much deadlier outcome. Furthermore, although, MPXV is usually resolved by itself, the current international outbreak showed that it might be life-threatening as well (106). Indeed, MPXV belongs to biosafety level 3 pathogens according to the EU regulations and is categorized as a high-threat virus (109). Alarmingly, the COVID-19 pandemic still exists and infects hundreds of thousands of people daily which makes the world population more vulnerable to MPXV. The high mutation rate of MPXVs isolated during the recent outbreak (42) along with all the abovementioned indicates the urgent need for developing specific, universal MPXV vaccine candidates to ultimately control this emerging virus and be prepared for any sudden outbreak. Considering all the above-stated information along with the extremely advantageous characteristics of nucleic acid-based vaccines, developing a new, next-generation MPXV vaccine certainly makes sense. The efficacy, safety, and simple and rapid production will make the nucleic acid-based MPXV vaccine clinically and socio-economically valuable.

## Design of potentially universal MPXV vaccine based on conserved elements

Nucleic acid vaccines do not require a complicated manufacturing process as after the immunization, the body becomes a bioreactor of the viral antigen. Thus, the process of vaccine development is cell-free, simpler, cost and time-effective. Above all, these types of vaccines are favorably safe. In order to design a potentially universal vaccine that will be effective against MPXV, VARV, and VACV, making the multi-epitope vaccine based on the conserved elements of the reasonably selected antigens seems to be the excellent way. First, the antigens should be selected according to their function in viral infection. In case of MPXV, the antigens listed in **Table 1** seem to be reasonable targets.

**Table 1:** List of the potential target antigens of MPXV and their corresponding proteins in VARV and VACV for the nucleic acid vaccine design, their location, function, and characteristics.

Name in MPXV	Name in VARV	Name in VACV	Location	Function and characteristics	Ref.
A5L	A4L	A4L	IMV	Immunodominant virion core protein; Needed for the progression of the infection	(51, 110)
A29L	A30L	A27L	IMV	Surface membrane fusion protein; Binds to cell surface heparan; Neutralizing antibody target	(45, 51, 110)
A30L	A31L	A28L	IMV	Envelope protein; Virus entry into a host; Cell-cell fusion (syncytial formation); Neutralizing antibody target	(51, 111)
A35R	A36R	A33R	EEV	Envelope glycoprotein; Formation of actin-containing microvilli and cell-to-cell spread of virion; Neutralizing antibody target	(51)
B2R	J9R	A56	EEV	EEV membrane glycoprotein hemagglutinin; prevents cell fusion	(51, 111)
B6R	B7R	B5R	EEV	Palmitylated glycoprotein; Required for efficient cell spread; Complement control	(51)
C15L	C13L	F9L	IMV	Neutralizing antibody target	(51, 111)
H3L	I3L	H3L	IMV	Surface protein; Binds heparin and cell surface proteoglycans	(51, 111)
M1R	M1R	L1R	IMV	Myristylated surface membrane protein; Virus entry into a host; Neutralizing antibody target	(51)

**Figure 5:**
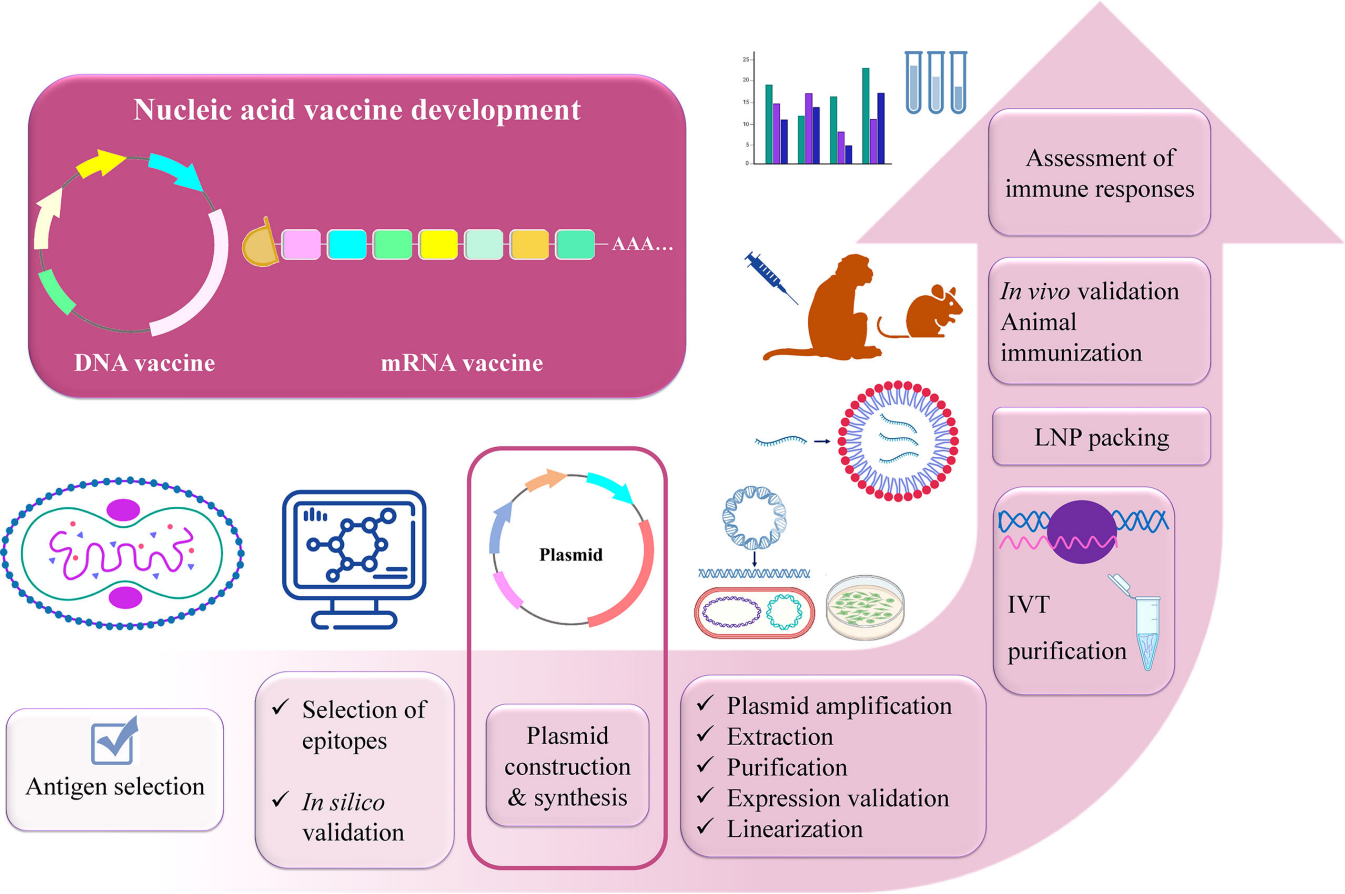
Outline of designing universal multi-epitope vaccine against MPXV, VARV, and VACV.

After selecting the antigens, the conserved sequences of the selected viral proteins should be determined *via* immunoinformatics tools (112–114). Luckily, there is a number of immunoinformatics approaches that can be used. The experimentally tested epitopes containing these conserved sequences can be found or predicted on the immune epitope database (IEDB) (115). After the final mRNA or DNA construct is designed using the preferable epitopes and optimized *via* selecting certain 5’ and 3’ untranslated regions (UTRs), proper linkers, and immune-modulator adjuvants (116), again immunoinformatics analyses should be conducted such as prediction of vaccine structure, immunogenicity, protectiveness, allergenicity, physicochemical properties, receptor-binding capacity, immune response caused *via* immune simulation, etc. (117–130). This will save time to anticipate the potential outcome of the designed vaccine. Once the results are favorable, the *in vitro* and *in vivo* studies will eventually validate the protectiveness of a potentially universal vaccine against MPXV. For the experimental validation, the following steps are proposed to be done: plasmid DNA expressing the gene of interest is synthesized and transformed into DH5α competent *E. coli* strain for amplification. It is followed by the extraction of plasmids from bacterial cells and purification. When the successful protein expression is confirmed *via* mammalian cell transfection, the plasmid can be used as a DNA vaccine for further *in vivo* studies or it should be linearized and *in vitro* transcribed into mRNA. mRNA is capped (5’-cap) for stability, protection from degradation, and facilitation of ribosomal recruitment (94). After mRNA is purified, its expression levels should be tested *via* cell transfection that can be followed by encapsulation with the proper delivery system such as lipid nanoparticles (LNPs) (28) and animal immunization experiments can be proceeded. Cellular and humoral immune responses elicited by the mRNA vaccine and the protection after viral challenges should be evaluated with the proper analyses. The outline of the development of a universal nucleic acid vaccine against MPXV is illustrated in **Figure 5**. In terms of mRNA vaccine side effects, it mainly depends on the dosage. Remarkably, this problem might be solved with self-amplifying (saRNA) or trans-amplifying RNA (taRNA) vaccine development that represents the next-generation nucleic acid vaccines owing to their lower dosage requirements. E.g., Vogel et al. demonstrated that 64-fold less material is needed for inducing the same immunity with saRNA compared to conventional mRNA vaccine against influenza virus (131). Compared with the mRNA molecule (~2000 nt), saRNA is longer (~10,000 nt) as besides the gene of interest, it contains the viral replicase genes which is based on the four non-structural proteins (nsPs) of alphaviruses. The presence of nsPs replicon, allows the molecule to be self-amplified in the host cell, producing the great number of desired antigens (132, 133). The viral replicase replicates the entire RNA as well as the sub-genomic RNA (133). As a result, higher and long-lasting antigen expression takes place. Remarkably, the challenge of saRNA large size can be dealt with using taRNA. The taRNA technology denotes splitting the saRNA molecule into two shorter molecules– encoding replicase and gene of interest separately (133, 134).

## Summary

Even though MPXV is not characterized as highly contagious virus as SARS-CoV-2, and smallpox vaccine that is effective against MPXV is available, attention should not be relaxed on developing specific vaccine candidates and seeking treatment ways for this virus. Unequivocally, it is much better to prevent disease in healthy populations than to make an effort to treat disease in already sick patients. The realignment of vaccination strategies as proposed here will work for the common well-being of the human population, particularly for the vulnerable population or those who have close contact with animals such as monkeys or rodents. It is also important that lately MPX was identified in a pet dog which worsens the situation meaning that the virus might circulate in animals and impede its eradication. Here, we provide the rationale for a potentially universal immunization strategy for multi-epitope nucleic acid-based vaccine design. The proposed vaccine construction is based on the conserved epitopes that gives the basis of its potential universality among newly formed mutated strains of MPXVs as well as strengthening the immunity against VARV, and VACV. Thus, the proposed strategy may be one step forward to speeding up overcoming the current outbreak as well as preventing other potential outbreaks.

## Author contributions

DP, NR, and YH contributed to the study conceptualization, DP and NR contributed equally to the drafting and editing of the work, CL revised the manuscript, XW contributed to preparing a figure, YH and PGW supervised. All authors contributed to the article and approved the submitted version.

## Funding

This work was supported by the Shenzhen Science and Technology Innovation Program (KQTD20200909113758004).

## Acknowledgments

Some parts of the figures were created with BioRender.com.

## Conflict of interest

The authors declare that the research was conducted in the absence of any commercial or financial relationships that could be construed as a potential conflict of interest.

## Publisher’s note

All claims expressed in this article are solely those of the authors and do not necessarily represent those of their affiliated organizations, or those of the publisher, the editors and the reviewers. Any product that may be evaluated in this article, or claim that may be made by its manufacturer, is not guaranteed or endorsed by the publisher.
